# Patriotism's Impact on Cooperation with the State: An Experimental Study on Tax Compliance

**DOI:** 10.1111/pops.12294

**Published:** 2015-08-28

**Authors:** Katharina Gangl, Benno Torgler, Erich Kirchler

**Affiliations:** ^1^ University of Vienna, Zeppelin University; ^2^ Queensland University of Technology, CREMA – Center for Research in Economics, Management and the Arts; ^3^ University of Vienna

**Keywords:** national pride, tax compliance, trust, voluntary cooperation, social capital

## Abstract

Although it seems reasonable to assume that activating patriotism might motivate citizens to cooperate with the state in reaching societal goals, the empirical evidence supporting this contention is based mostly on correlational rather than experimental studies. In addition, little is known on whether patriotism can be manipulated without simultaneously triggering nationalism and on the psychological processes which determine the patriotism‐cooperation relation. This current article reports results of one survey and three experiments that manipulate patriotism by displaying either a national flag or national landscapes or by priming national achievements. The outcomes indicate that reported and manipulated patriotism indirectly increase tax compliance, although the national flag also increases nationalism. National achievements, on the other hand, seemingly increases trust in national public institutions and the voluntary motivation to cooperate, whereas national landscapes only increase the voluntary motivation to cooperate. Hence, it is possible to increase social capital in the form of trust and cooperation through patriotism without fostering nationalism as well.

The state and its public institutions depend on such citizen cooperation as tax compliance, which might potentially be increased by using patriotism as a promotional tool (Konrad & Qari, 2012; Mummendey, Klink, & Brown, 2001). Public information campaigns, brochures, or websites, for instance, might use the national flag or its colors to foster citizens’ identification with their community and thus their willingness to cooperate (Jones, 1996; McMakin, Malone, & Lundgren, 2002). Survey studies do in fact report a positive relation between patriotism and prosocial behavior such as tax compliance, election participation, or blood donation (Huddy & Khatib, 2007; Wenzel, 2007). Surprisingly, however, no experiments were conducted showing the positive impact of patriotism on cooperation. Although there are several experiments analyzing whether national symbols such as the flag generate patriotism rather than destructive nationalism (e.g., Becker, Enders‐Comberg, Wagner, Christ, & Butz, 2012; Butz, Plant, & Doerr, 2007; Hassin, Ferguson, Shidlovski, & Gross, 2007; Kemmelmeier & Winter, 2008), only few experiments have empirically tested the effects of other potentially promotional tools of patriotism like national achievements (Mummendey et al. 2001) and national landscapes. There is also a lack of empirical studies on the psychological processes through which patriotism might impact cooperation indirectly by generating trust in public institutions and voluntary motivation to cooperate (Kirchler, Hoelzl, & Wahl, 2008; Wenzel, 2002). Yet insights into the effects of promotional patriotic tools would not only enhance theoretical understanding of patriotism and its effects but might also allow public institutions to choose the most effective communication instruments for enhancing cooperation of citizens by patriotism and not nationalism.

This present study, therefore, uses the results of one survey and three experiments to test for an effect of three common patriotism promoting tools—the national flag, national achievements, and national landscapes—on cooperation with the state in the form of tax compliance. It also assesses whether the impact of such tools on tax cooperation is direct or indirect via increased trust in tax authorities and voluntary motivation to cooperate.

## Theoretical Background and Research Questions

One useful explanation of patriotism and its effects is offered by social identity theory (Tajfel, 1974; Tajfel, Billig, & Bundy, 1971), which may describe patriotism in terms of citizen self‐categorization as members of a specific social group such as a national or local community (Tajfel & Turner, 1986). Such self‐categorization provides citizens with a positive self‐concept through such positive emotions as love and pride in national achievements (Federico, Golec, and Dial, 2005; Tajfel & Turner, 1986), meaning that patriotism can be more narrowly defined as “positive identification and feelings of affective attachment to one's country” (Schatz, Staub, & Lavine, 1999, p. 152). This patriotism as social identity provides important guidance for social behavior (Huddy & Khatib, 2007; Tajfel & Turner, 1986): individuals tend to copy the actions of others seen as members of their own social group (Cialdini & Goldstein, 2004) and likely increase their cooperation with them (Blader & Tyler, 2009; Tajfel, 1974; Terry, Hogg, & White, 1999). Nevertheless, this increased cooperation with individuals sharing the same identity is often accompanied by decreased cooperation with individuals perceived as belonging to an outgroup (Tajfel & Turner, 1986). For instance, shared identity can easily be manipulated experimentally by asking participants to express a preference for the paintings of either Paul Klee or Wassily Kandinsky (Tajfel et al., 1971). As a consequence, these participants tend to identify more with others that share their painter preference who they subsequently reward more and punish less than participants with a different painter preference. Participants in these experiments are also willing to maximizing the difference between ingroup and outgroup members at the price of deliberately disadvantaging the outgroup (Tajfel, 1974; Tajfel et al., 1971). Hence, increased cooperation with one's own group often goes along with deliberate discrimination of an outgroup.

Identification with one's own country and community can also be the basis of “healthy” patriotism, on the one hand, and “destructive” nationalism, on the other hand (Kosterman & Feshbach, 1989; Schatz et al., 1999). Patriotism reflects a psychological state of love for and pride in one's country and its community, whereas nationalism reflects a belief that one's own country should dominate and discriminate against other countries and their citizens (Federico et al., 2005; Kosterman & Feshbach, 1989; Mummendey et al., 2001). Patriotism, in principle based on positive evaluations of the own nation, however, does not exclude or even demand criticism of the own community if the community does not meet certain standards (Federico et al., 2005; Schatz et al., 1999). In contrast, nationalism can be characterized as a form of ethnocentrism in which the own community is not criticized and perceived as homogeneous—a view, typically combined with hostility towards foreigners living in one's own country (Blank & Schmidt, 2003; Federico et al., 2005).

Patriotism and nationalism are connected (Blank & Schmidt, 2003). However, the two are not necessarily reciprocally related (Brewer, 1999) and hence, might be activated independently from each other (Mummendey et al., 2001). According to experimental survey data from several countries, patriotism is activated by comparing one's own country's present with its past or by evaluating it without any explicit comparative standard or rational explanation (Mummendey et al., 2001). In contrast, nationalism tends to be activated when individuals are asked to compare their own country with other countries. These studies also show that thinking about national achievements as a comparison between past and present compared to thinking about differences between the own country and other countries does not lead to derogation of outgroup members like foreigners (Mummendey et al., 2001). Analogously, although no empirical evidence is yet available, the ability of identification with the local community versus the identification with the national community also may render it capable of reducing nationalistic tendencies (Herrmann & Brewer, 2004; Paasi, 2003). A reason might be that local patriotism in contrast to national patriotism is more dynamic concerning historical and geographical development (Raagmaa, 2010), a characteristic which might prevent immutable views and discrimination of outgroup members.

Identification with the community in one's country or local region in the form of patriotism, therefore, unlike nationalism, is social capital that fosters important prosocial behavior (Bar‐Tal, 1993; Raagmaa, 2010; Rothstein, 2003) and thereby fuels social prosperity (Rothstein, 2003). For example, an analysis of World Value Survey (WVS) data from 45 countries suggests that those who are proud to be members of their own community show more trust in other people and contribute to a society's social capital by such responsible behaviors as not claiming unjustified government benefits, not buying stolen goods, and not lying in their own interests (Whiteley, 1999). Other studies also associate identification with one's own community with donation of blood or money and attentiveness to elections (Huddy & Khatib, 2007; Skitka, 2005). For tax compliance particularly, surveys from across the globe indicate that national pride and identification with one's own country or local community, as measurements of national and local patriotism, are positively associated with high tax morale (Hartner, Rechberger, Kirchler, & Wenzel, 2011; Konrad & Qari, 2012; Torgler, 2003, 2004, 2005; Torgler & Schneider, 2005). For instance, based on data from the WVS, European Value Survey (EVS), and Latinobarómetro, being proud to be a member of one’ s own country is positively correlated with tax compliance in Asia (Torgler, 2004), Central and Eastern Europe (Torgler, 2003), Austria (Torgler & Schneider, 2005), and Latin America (Torgler, 2005). A two‐wave study of 1,161 Australians also indicates that identification with the Australian community positively impacts social norms of tax compliance and tax‐compliance intention (Wenzel, 2005). Admittedly, the very large samples in these studies (e.g., 92,141 participants in Whiteley, 1999) increase the possibility that the patriotism–tax‐compliance relation is simply an artefact of large sample size (Lin, Lucas, & Shmueli, 2013); however, the effect sizes are not only statistically meaningful but also economically significant. Nevertheless, no experimental evidence exists for a causal impact of patriotism on cooperation, and none of the existing studies analyze the psychological processes that might mediate the relation between patriotism and tax compliance.

Concerning psychological processes which might cause indirect effects of patriotism, the slippery slope framework of tax compliance assumes that identification with one's own community should impact tax compliance through increased trust in the tax authority that then engenders voluntary tax cooperation (Gangl, Hofmann, & Kirchler, 2015; Kirchler et al., 2008). That is, the more citizens identify with their state and community, the more they should trust state authorities seeing them as benevolent and dedicated to the common good, and the more citizens should be motivated to voluntarily pay their taxes (Kirchler et al., 2008). Both experimental and nonexperimental research suggests that a shared identity leads to an evaluation of others as trustworthy (Tyler, 2001; Voci, 2006) and indicates that trust in tax authorities fuels individual voluntary motivation and willingness to pay them (Kogler et al. 2013; Wahl, Kastlunger, & Kirchler, 2010). Hence, based on both the slippery slope framework and existing empirical data, patriotism's impact is likely to be based on an indirect psychological process that fuels trust in the tax authorities, generates voluntary motivation to cooperate, and thereby increases cooperation.

Despite these valuable insights, however, no empirical studies have as yet examined whether patriotism impacts cooperation directly or indirectly through different evaluative, emotional, and ultimately motivational psychological processes (Bar‐Tal, 1993; Wenzel, 2007). A direct impact of patriotism would provide evidence that patriotisms’ effect is predominantly implicit and emotional, conforming with a definition of patriotism as an emotional affiliation to one's country (Bar‐Tal, 1993; Schatz & Lavine, 2007; Schatz et al., 1999). In contrast, an indirect effect via deliberate evaluations on whether or not to trust public institutions would suggest that patriotism has a deliberative cognitive impact on individuals as they evaluate whether or not to trust state institutions and only then develop a voluntary motivation that leads them to cooperate (Gawronski & Bodenhause, 2003; Wenzel, 2002; Wenzel & Jobling, 2006). At present, little is also known about whether the way that patriotism is activated, for instance, whether national patriotism or local patriotism is triggered, generates different processes such that some promotional patriotic tools induce a direct emotional process leading to automatic loyalty and cooperation whereas others lead to an indirect deliberate process that results in reason‐based motivation to support one's own community.

The present study analyses the results of one survey and three experiments to determine whether typical patriotism promoting tools like the national flag, national achievements, and national landscapes lead to patriotism or nationalism. It also tests whether these tools impact tax cooperation and whether this effect is direct or indirect, mediated by reason‐based trust in tax authorities and voluntary motivation to cooperate. To ensure results with high external validity, the study data were collected from field settings in which government institutions would typically use promotional tools to promote tax compliance—for example, the business sections of newspapers.

The remainder of the article is organized as follows: the next section outlines the survey results, after which we report the outcomes of the three experiments on the effects of the national flag, national achievements, and national landscapes. We then discuss the theoretical and practical implications of these findings and explore how patriotism can be used to increase social capital and citizens’ cooperation with the state.

## Survey

### Sample

Our sample was made up of 84 Austrians, the majority of whom were male (66.7%), with an average age of 44.13 (*SD* = 12.24, range: 23–72). Most participants had a university degree (33.3%), were employed in the private sector (45.2%), and reported a monthly income between 1,501 and 3,000 Euro (40.5%). When asked to indicate their experience with tax authorities on a 7‐point scale, participants rated themselves overall as rather experienced (*M* = 4.45, *SD* = 1.67).

### Material

The online questionnaire included items on patriotism, nationalism, trust in the tax authorities, voluntary cooperation, and tax‐compliance intention. Patriotism was assessed as national and local patriotism, based on national pride and identification with the local community. National pride was measured on a 7‐point Likert scale from 1 = *not proud at all* to 7 = *very proud*, using the following WVS item: “How proud are you to be Austrian?” Identification with the local community “I see myself as a part of my local community” was measured on a 7‐point Likert scale from 1 = *totally disagree* to 7 = *totally agree*, again based on the corresponding WVS item. Nationalism was measured based on four items from Meier‐Pesti and Kirchler's (2003) nationalism scale: (1) “It is important that Austria is different than other countries”; (2) “Austria is a better country than most other countries”; (3) “It would be good if Austria would differentiate itself more from other countries”; and (4) “It is justified that Austria is better off than other countries.”

Trust in tax authorities was measured using five items modelled after Castelfranchi and Falcone's (2010) sociocognitive definition of trust which captures trust as a reason‐based and deliberate decision to trust: “I trust the tax authorities in Austria … (1) because of their highly motivated employees who give comprehensive advice, (2) because they involve no financial dangers for me, (3) because they have the important objective of giving competent advice, (4) because they benevolently advise taxpayers, and (5) because they give me competent advice.” Voluntary tax cooperation was assessed using five items from the TAX inventory (Kirchler & Wahl, 2010): “When I pay my taxes as required by the regulation, I do so … (1) because to me it's obvious that this is what you do, (2) to support the state and other citizens, (3) because I like to contribute to everyone's good, (4) because for me it's the natural thing to do, and (5) because I regard it as my duty as a citizen.” All scales were measured on a 7‐point Likert scale from 1 = *I do not agree* to 7 = *I totally agree* and are highly reliable, with a Cronbach's α equal to .79, .91, and .88, respectively.

Tax‐compliance intention was assessed based on the following scenario: Participants were asked to imagine having to pay 40% tax on a self‐employed income of 83,330 Euro with an audit probability of 17% and a tax evasion fine equal to the amount of the tax evaded. An open‐ended question then asked them to indicate how much tax they would be willing to pay in this scenario. The questionnaire also collected sociodemographic data, including citizenship, sex, age, education, occupation, income, and experience with tax authorities.

### Procedure

To recruit participants, we posted an invitation to take part in a survey on the financial decision making of individuals currently living in Austria on the business discussion forums of eight Austrian newspapers. Those who followed the link were redirected to an online questionnaire structured in four sections: The first asked participants to indicate their citizenship. The second included the scenario measuring tax‐compliance intentions, followed by items on national pride, identification with the local community, nationalism, trust in tax authorities, and voluntary cooperation. The third and final sections collected the sociodemographic characteristics, after which participants were given an opportunity to leave comments and were thanked for their participation.

### Results and Discussion

To identify the overall relation between national pride, identification with the local community, nationalism, trust, voluntary cooperation, and tax compliance, we conducted individual‐level correlational analyses to assess three factors: whether national pride and nationalism are related to trust and voluntary cooperation, whether identification with the local community is related to voluntary cooperation, and whether voluntary cooperation is related to tax‐compliance intention. The results, reported in Table 1, show no direct link between national pride, identification with the local community, nationalism, and tax compliance.

**Table 1:** Intercorrelation of Scales and Items of the Survey

	*M* (*SD*)	1	2	3	4	5
1. National pride	5.16 (1.79)					
2. Identification with local community	4.94 (1.95)	.07				
3. Nationalism	4.31 (1.49)	.61***	−.00			
4. Trust	3.52 (1.41)	.28*	.15	.20†		
5. Voluntary cooperation	5.13 (1.51)	.25*	.33**	.01	.32**	
6. Tax‐compliance intention	81.58% (29.25)	.14	.00	.01	.14	.29*

*Note. M* = mean, *SD* = standard deviation

†*p* < .10; *p < .05; **p < .01; ***p < .001

To test the assumption that national pride, identification with the local community, or nationalism have an indirect effect on tax compliance via trust and voluntary cooperation, we used Process software (Hayes, Preacher, & Myers, 2011) to run simple and multivariate linear regressions on a bootstrap sample of 1,000 participants. This analysis assessed the extent to which the relation between two variables might be indirect through a path of other mediating triggers (Hayes, 2013; Hayes et al., 2011).

The results indicate that, as expected, national pride leads to trust (β = .28, *p* = .01) which impacts voluntary cooperation (β = .26, *p* = .04), and in turn impacts tax compliance (β = .28, *p* = .03; 95% CI [0.05; 1.43]. The indirect effect via pride and trust alone (95% CI [−1.22; 1.60]) or pride and voluntary cooperation alone (95% CI [−0.14; 3.06]), however, is not significant. The results for identification with the local community show that identification with the local community triggers voluntary cooperation (β = .33, *p* = .006), which in turn impacts tax compliance (β = .29, *p* = .03; 95% CI [0.03; 3.94]). Other indirect paths are not significant (identification and trust: 95% CI [−0.49; 1.13]) or marginally not significant (identification, trust, and voluntary cooperation: 95% CI [−0.00; 1.01]). Nationalism seems also to be indirectly connected to tax compliance: it triggers trust in tax authorities (β = .20, *p* = .09), which fosters voluntary cooperation (β = .32, *p* = .009), and in turn tax compliance (β = .28, *p* = .03; 95% CI [0.02; 1.31]). The other indirect effects are not significant (nationalism and trust: 95% CI [−0.71; 2.16]; nationalism and voluntary cooperation: 95% CI [−1.99; 1.00]). In sum, we identify only indirect relations between identification with the community and tax‐compliance intention: national pride and nationalism are related to trust in tax authorities, which fuels voluntary motivation to cooperate and in turn tax compliance. Identification with the local community, in contrast, seems to be related to voluntary cooperation (and less to reason‐based trust), which fuels tax compliance. Hence, whereas national pride and nationalism tend to trigger deliberate processes in which evaluations about whether or not to trust tax authorities play a role, identification with the local community seems to be a rather emotional process that fuels patriotic motivations based on civic duty and not deliberate evaluation whether or not tax authorities can be trusted.

## First Experiment: National Flag

### Sample

The first experimental sample consisted of 110 Austrians, the majority of them male (70.9%), with an average age of 41.44 (*SD* = 12.18). Most participants had a university degree (34.5%), were employed in the private sector (36.4%), and had a monthly income of between 1,501 and 3,000 Euro. Again, when asked on a 7‐point scale about their experience with the tax authorities, participants rated themselves overall as rather experienced (*M* = 4.28, *SD* = 1.82).

### Material and Procedure

The recruitment of participants, conducted in online newspaper forums, was identical to that for the survey. After participants indicated their citizenship, their patriotism was manipulated by randomly assigning them to one of two conditions: an image of their national flag or an image of a fictitious flag (see the online supporting information). Next, participants were presented with the same tax‐compliance scenario as in the survey and filled in the same items on national pride, identification with the local community, nationalism (Cronbachs’ α = .80), trust in tax authorities (Cronbachs’ α = .92), voluntary cooperation (Cronbachs’ α = .92), and sociodemographic characteristics.

### Results and Discussion

The differences between the experimental groups were identified using *t*‐tests, with the national versus fictitious flag as the independent variable and national pride, identification with the local community, nationalism, trust, voluntary cooperation, and tax‐compliance intention as dependent variables. The national flag led to greater national pride than the fictitious flag (*t*(101) = 2.31, *p* = .02, Cohen's *d* = .47) and also to marginally more nationalism (*t*(98) = 1.76, *p* = .08, Cohen's *d* = .35). No differences emerged for identification with the local community (*t*(102) = 1.15, *p* = .25). The results also indicate, however, that the national flag induced no higher trust in tax authorities (*t*(90) = 0.65, *p* = .52), tax‐compliance intentions (t(108) = 1.14, *p* = .26), or voluntary cooperation (t(84) = 1.56, *p* = .12) than the fictitious flag. Respective means and standard deviations of the experimental groups are displayed in Table 2.

**Table 2:** Means and Standard Deviations of the National Flag Experiment

	National Flag	Fictitious Flag
	*M* (*SD*)	*M* (*SD*)
National pride	5.32 (1.45)	4.48 (2.10)
Identification with local community	5.02 (1.93)	4.63 (1.59)
Nationalism	4.24 (1.57)	3.71 (1.44)
Trust in the tax authorities	3.22 (1.45)	3.02 (1.41)
Voluntary cooperation	5.36 (1.74)	4.78 (1.71)
Tax‐compliance intention	79.22% (26.49)	72.37% (34.80)

*Note. M* = mean, *SD* = standard deviation

The corresponding regression analyses, again on a bootstrap sample of 1,000 participants, indicate an indirect effect. The national flag elicits national pride (β = −.22, *p* = .02), which in turn impacts tax compliance (β = .27, *p* = .009), 95% CI [−13.71, −0.58]. The results also show that national pride leads to more reason‐based trust in tax authorities (β = .27, *p* = .01) and in turn fosters voluntary cooperation (β = .34, *p* = .002) and tax compliance (β = .28, *p* = .01; 95% CI [−2.09,−0.07]). The fact that no path without national pride is significant (trust in tax authorities: 95% CI [−2.39, 1.16]; voluntary cooperation: 95% CI [−10.78, 0.77]) implies that the national flag increases intended tax compliance indirectly through an explicit and deliberative process based on trust in tax authorities. Given the *t*‐test result of no impact for the national flag on identification with the local community, no indirect effect via identification with the local community is possible. Also only tendencies but no significant indirect effects emerge for elicited nationalism (nationalism: 95% CI [−4.10, 4.20]; nationalism and trust: 95% CI [−2.20, 0.25]; nationalism and voluntary cooperation: 95% CI [−0.05, 3.87]; nationalism, trust, and voluntary cooperation: 95% CI [−1.70, 0.01]). These results indicate that an indirect effect similar to that found in the survey study occurs only if the national flag impacts national pride thereby indirectly fostering trust and voluntary cooperation and in turn tax‐compliance intention. No such indirect effect on tax compliance is observed if the flag impacts nationalism.

## Second Experiment: Priming

### Sample

Our sample comprised 99 Austrians, the majority of whom were male (63.6%), with an average age of 43.09 (*SD* = 12.20, range: 17–71). Most participants had a university degree (40.4%), were employed in the private sector (38.4%), and reported a monthly income between 1,501 and 3,000 Euro (40.4%). When asked to indicate their experience with tax authorities on a 7‐point scale, participants rated themselves overall as rather experienced (*M* = 4.46, *SD* = 1.92).

### Material and Procedure

The material, procedure, and analytical strategy were almost identical to those in the flag experiment except that instead of using a flag to manipulate patriotism, we employed national achievements as a priming mechanism to trigger patriotism (cf. Mummendey et al., 2001). Specifically, participants were asked to write down three reasons why they like (dislike) living in Austria, a positive (negative) priming eliciting high (low) patriotism. They were then instructed to read a short text highlighting recent Austrian achievements or shortcomings in such areas as health care, infrastructure, the quality of democratic institutions, and the economy. Next, as in the survey and experiment 1, participants were presented with a tax‐compliance scenario and items on national pride, identification with the local community, nationalism (Cronbach's α = .80), trust in the tax authorities (Cronbach's α = .89), voluntary cooperation (Cronbach's α = .93), and sociodemographic characteristics.

### Results and Discussion

The differences between experimental groups were again identified using *t*‐tests with positive versus negative priming as the independent variable and national pride, identification with the local community, nationalism, trust, voluntary cooperation, and tax‐compliance intention as dependent variables. The positive priming for Austria led to marginally more national pride than the negative priming (*t*(92) = 1.84, *p* = .07, Cohen's *d* = .39), but no differences are observable for identification with the local community (*t*(92) = 1.06, *p* = .29) or nationalism (*t*(91) = −1.00, *p* = .32). Positive priming also generated more trust in tax authorities (*t*(97) = 1.89, *p* = .01, Cohen's *d* = .57) than did negative priming and also lead to marginally more tax‐compliance intention (*F*(97,1) = 3.58, *p* = .06, Cohen's *d* = .38). No differences emerge, however, for voluntary cooperation (*t*(81) = 0.90, *p* = .37). Respective means and standard deviations of the experimental groups are illustrated in Table 3.

**Table 3:** Means and Standard Deviations of the Priming Experiment

	Positive Priming	Negative Priming
	*M* (*SD*)	*M* (*SD*)
National pride	5.08 (1.75)	4.35 (2.03)
Identification with local community	5.15 (1.95)	4.68 (2.29)
Nationalism	4.10 (1.55)	4.45 (1.74)
Trust in the tax authorities	3.64 (1.50)	2.83 (1.36)
Voluntary cooperation	5.22 (1.81)	4.84 (1.88)
Tax‐compliance intention	81.77% (28.23)	69.43% (36.16)

*Note. M* = mean, *SD* = standard deviation

To identify the underlying psychological mechanisms, we again tested for indirect effects using a bootstrap sample of 1,000 participants. The results indicate that the priming impacts national pride (β = −.19, *p* = .07) which influences trust (β = .15, *p* = .15) and in turn impacts voluntary motivation to cooperate (β = .41, *p* < .001) and thus, tax compliance (β = .65, *p* < .001; 95% CI [−4.04; −0.01]). The other paths with national pride are not significant (national pride: 95% CI [−9.76; 0.16], national pride and trust in tax authorities: 95% CI [−0.01; 2.49]). However, the analysis also suggests paths without national pride: Compared to negative priming, positive priming leads to more trust in tax authorities (β = −.26, *p* = .01) which in turn impacts tax compliance (β = .16, *p* =16; 95% CI [0.25; 9.09]) or impacts first voluntary cooperation (β = .43, *p* < .001) and then tax‐compliance intention (β = .68, *p* < .001; 95% CI [−10.80; −0.89]). Likewise, the *t*‐test results indicate that priming has no impact on identification with the local community or nationalism, meaning that no indirect effect is possible via identification or nationalism. Taken together, these findings indicate that priming increases national pride and in turn, similar to the survey study, trust, voluntary motivation to cooperate, and tax compliance. However, the present results also suggest that reason‐based trust in tax authorities seems to be particularly important for the indirect impact of the priming on tax compliance. Thus, the priming of national achievements seems to increase tax‐compliance intention through an explicit deliberative process.

## Third Experiment: National Landscapes

### Sample

The third experimental sample consisted of 74 Austrians, the majority male (91.9%), with an average age of 43.14 (*SD* = 13.02, range: 21–70). As before, most participants had a university degree (40.5%), were employed in the private economy (37.8%), and earned between 1,500 and 3,000 Euro (55.4%) a month. When asked on a 7‐point scale about their experience with the tax authorities, participants again rated themselves overall as rather experienced (*M* = 4.55, *SD* = 1.79).

### Material and Procedure

Although patriotism was manipulated differently, the material and procedure in this intervention were identical to those in the previous experiments. Because market research suggests that national landscapes are the most important reason for Austrians to be proud of their country (Beutelmeyer, 2011), participants were randomly assigned either to a condition of seeing images of Austrian landscapes or a control condition of seeing images of Australian landscapes. For both conditions, participants were randomly presented with four pictures in two different orders to avoid order effects (see the supporting information for the images).

As in the national flag experiment and priming task, the dependent variables were tax‐compliance intention, national pride, identification with the local community, nationalism (Cronbachs’ α = .83), trust in tax authorities (Cronbachs’ α = .83), voluntary cooperation (Cronbachs’ α = .89), and sociodemographic characteristics. Participants were also asked to indicate whether they recognized the landscapes presented and to write down what they thought each picture showed. Based on this information, we were able to calculate a *recognition* variable indicating whether participants (1) did not recognize or (2) recognized the landscape shown.

### Results and Discussion

The differences between experimental groups were identified using ANCOVA, with national versus foreign landscape as the independent variable; national pride, identification with the local community, nationalism, trust, voluntary cooperation, and tax compliance as dependent variables; and recognition as the control variable. Although the national landscape pictures led to more identification with the local community (*F*(71,1) = 3.93, *p* = .05, η^2^ = .05), the national (Austrian) landscape pictures did not generate more national pride (*F*(71,1) = 0.14, *p* = .71) or nationalism (*F*(71,1) = 1.02, *p* = .32) than the foreign (Australian) landscape controls. The results also reveal no differences between the conditions for trust in tax authorities (*F*(71,1) = 0.29, *p* = .60) or voluntary cooperation (*F*(71,1) = 1.68, *p* = .20). They do, however, indicate that national landscapes led to higher tax‐compliance intention than foreign landscapes (*F*(71,1) = 5.56, *p* = .02, η^2^= .07). Respective means and standard deviations of the experimental groups are shown in Table 4.

**Table 4:** Means and Standard Deviations of the National Landscapes Experiment

	National Landscape	Foreign Landscape
	*M* (*SD*)	*M* (*SD*)
National pride	4.81 (2.03)	4.66 (1.58)
Identification with local community	4.92 (1.73)	4.03 (2.01)
Nationalism	3.91 (1.65)	4.23 (1.47)
Trust in the tax authorities	2.94 (1.31)	2.82 (1.13)
Voluntary cooperation	5.11 (1.49)	4.88 (1.66)
Tax‐compliance intention	86.25% (23.07)	76.92% (26.42)

*Note. M* = mean, *SD* = standard deviation

The significant recognition covariate for tax‐compliance intention (*F*(71,1) = 8.58, *p* = .01, η^2^= .11) also indicates that nonrecognition increases tax‐compliance intention (Spearman correlation = −.29, *p* = .01) in a similar manner in both experimental conditions (national landscape: Spearman correlation = −.30, *p* = .07; foreign landscape: Spearman correlation = −.41, *p* = .01). The covariate is insignificant, however, for pride, identification with the local community, nationalism, trust, and voluntary cooperation (all at *p* ≥ .10).

An additional analysis with recognition as a control variable was aimed at identifying the mechanisms determining the landscapes’ impact on tax compliance. This analysis, again conducted on a bootstrap sample of 1,000, points to an indirect impact via identification with the local community (β = −.24, *p* = .05), which impacts voluntary tax cooperation (β = .41, *p* < .001) and in turn tax‐compliance intention (β = .29, *p* = .02; 95% CI [−5.04; −0.01]). No other mediation models produced significant results: identification with the local community (95% CI [−3.34, 4.26]), identification with the local community and trust in tax authorities (95% CI [−2.40; 0.06]), trust in tax authorities (95% CI [−3.94, 1.99]), trust and voluntary cooperation (95% CI [−1.35; 0.43]), or via voluntary cooperation (95% CI [−6.34; 1.95]). According to the ANCOVA results, the landscapes have no impact on national pride or nationalism and can thus have no indirect effect via pride or nationalism. These findings imply that the landscapes’ impact on tax‐compliance intention is indirect and rather implicit. This assumption is also supported by the finding that the landscapes particularly increased tax‐compliance intentions of those participants who did not deliberately recognize them. As with the survey results, identification with the local community triggers the voluntary motivation to cooperate and in turn tax‐compliance intention without rational consideration of whether national tax authorities can be trusted.

## General Discussion and Conclusion

To demonstrate that patriotism can be used as a promotional tool for cooperation with the state, this article reports evidence from one survey and three experimental studies showing that certain patriotic materials can indeed impact identification with the community and increase cooperation. Nevertheless, the present article also shows that attempts to manipulate patriotism can also lead to destructive nationalism and apparently have different underlying mechanisms depending on whether the identification is with the national or regional community (Bar‐Tal, 1993; Mummendey et al., 2001). Specifically, our results indicate similar to previous research from the United States (Butz et al., 2007; Kemmelmeier & Winter, 2008), that the national flag, although it does have an impact on patriotism, also increases nationalism. National achievements (Mummendey et al., 2001) or national landscapes, on the other hand, impact patriotism without triggering nationalism. The results also show that whereas the processes underlying cooperation generated by identification with the national community include deliberate trust in national tax authorities, those underlying identification with the local community do not include trust in national authorities. The present article is one of the few which uses different priming methods to examine the impact of patriotism and nationalism on domestic policy attitudes.

The current findings confirm and expand research showing a connection between patriotism and cooperation with the state (Konrad & Qari, 2012; Whiteley, 1999). In particular, the present studies demonstrate that identification with one's own country or local community are indeed related to tax compliance but through indirect paths mirroring different psychological processes. Whereas identification with the community of one's own country (e.g., manipulated through the national flag or national achievements) impacts trust in national tax authorities, voluntary motivation to cooperate, and thus tax compliance, identification with the local community (e.g., manipulated through national landscapes) seems to impact tax compliance only via the voluntary motivation to cooperate out of a felt loyalty and not via deliberate trust in national authorities. However, identification with the local community might also involve trust in authorities, but in local authorities and not in national authorities. Future empirical studies are needed to replicate the present findings and additionally assess trust in local authorities.

The present results also indicate that promotional tools which trigger identification on a local level (e.g., through national landscapes) compared to identification on the national level (e.g., through the national flag) run a smaller risk of triggering nationalism next to patriotism (Herrmann & Brewer, 2004; Raagmaa, 2010). This might be related to the way local identity was manipulated in the present study. If local identity is triggered through local symbols such as flags or traditional costumes, a local identity might also increase nationalism as degradation of outgroup members.

While national flags and national achievements trigger patriotism, only national achievements seem to be able to trigger patriotism without nationalism. This outcome confirms previous experiments on the effect of national flags (e.g., Butz et al., 2007; Kemmelmeier & Winter, 2008) and national achievements (Mummendey et al., 2001). These results clearly suggest that public institutions should rather present national achievements than the national flag to increase cooperation of citizens. However, national achievements might also lead to nationalism if they are not carefully designed. For instance, if the comparison between ones’ country's past with its present leads to the impression that the country is more successful in mastering challenges than other countries, nationalism might be triggered as well. Therefore it can be suggested to public institutions to run pretests of promotional material they would like to use to ensure that their material is only eliciting patriotism and not nationalism.

The results on the covariate recognition of landscapes show that nonrecognition of landscapes leads to more tax‐compliance intentions than recognition of landscapes. Although this relation can be found in both experimental conditions (the national and the foreign landscape condition), it is only significant in the foreign landscape condition. Concerning the foreign landscapes, it is perhaps not surprising that explicit recognition of a foreign country is negatively related to national tax compliance. Nonrecognition of a foreign landscape might be perceived as a neutral landscape which does not influence tax‐compliance intentions. In contrast, the explicit recognition of a foreign country might lead to the impression that tax compliance does not support one's own community but a foreign community, a perception which might reduce tax‐compliance intentions. However, nonrecognition of national landscapes also tends to be related with more tax‐compliance intention than recognition of national landscapes. One could speculate that recognized national landscapes might be linked to concrete and sometimes negative attitudes which in turn reduce tax compliance. In contrast, nonrecognized national landscapes might unconsciously only be perceived as familiar and therefore might implicitly elicit more positive emotions and more tax‐compliance intentions. However, these are only speculations, and the underlying sample sizes necessitate approaching such arguments with caution. Future studies are needed to replicate the current finding and to shed light on the reasons why nonrecognition of landscapes might be positively related to cooperation with the state.

The present research is subject to certain limitations. First, although public institutions like tax authorities need the cooperation of foreign citizens, all participants in our research had Austrian citizenship. This choice was made based on the assumption that the Austrian national flag, national achievements, and/or national landscapes might not be as familiar to foreigners and might not, therefore, have equal power to increase their identification, trust, and cooperation. Nonetheless, future studies should investigate how identification with the country or region of residence can be triggered to increase trust and cooperation in foreigners.

Second, unlike the findings in previous research (Torgler, 2003; Whiteley, 1999), the survey results from this study identify no significant direct relation between patriotism and tax compliance, only a tendency for the two to be linked. We attribute this failure to our smaller sample sizes; had our survey used thousands of participants like others in the literature, this mere tendency might have grown in significance. Nonetheless, this result does underscore our experimental finding that the relation between patriotism and cooperation is not direct but indirect and mediated through trust and voluntary motivation to cooperate.

Finally, our research design does not take into account the fact that Austria has one of the highest tax morale and tax‐compliance rates across the globe (Alm & Torgler, 2006; Schneider, Buehn, & Montenegro, 2010). Future empirical research thus needs to clarify whether our findings can be replicated in countries with lower tax‐compliance rates and hence lower perceived social norms of compliance. Our findings do, however, emphasize the high relevance of patriotism for cooperation: patriotism seems to increase tax‐compliance intention even beyond Austria's already high tax‐compliance rates.

Despite these shortcomings, our findings have important theoretical and practical implications for governmental information campaigns promoting cooperation. First, they show that emphasizing national achievements not only promotes patriotism without nationalism but increases cooperation and maybe even more important, trust in public institutions. Given the declining levels of trust—and thus social capital—in Western democracies (Putnam, 1995), this outcome seems particularly important. Based on our findings, campaigns that make citizens aware of national progress can strengthen reason‐based trust and optimism, important determinants not only of tax compliance, but also of economic and social prosperity (Akerlof & Shiller, 2009; Gangl, Kastlunger, Kirchler, & Voracek, 2012). On the other hand, patriotic materials that trigger a local identity, although they too increase cooperation, may not have the important side‐effect of triggering trust in national public institutions. It also seems likely that a cooperation effect induced through an implicit cue like national landscapes may be short lived, whereas one induced through explicit positive evaluations of state achievements may have a long‐term impact. Although the use of national landscapes can be recommended to increase cooperation, public institutions that want to increase patriotism, trust, voluntary motivation to cooperate, as well as long‐term cooperation should particularly highlight national achievements in their promotional campaigns (Bar‐Tal, 1993; Mummendey et al., 2001).

Our observations also provide useful insights for public campaigns in general. Whereas some patriotic materials (e.g., the national flag) induce nationalism and should be avoided, others, such as national achievements or national landscapes, can increase citizens’ identification and cooperation with their community. Nevertheless, if a long‐term impact on patriotism, trust, and cooperation is pursued, campaigns should use salient and explicitly positive evaluations of state achievements (Bar‐Tal, 1993). Such an assumption is supported by our empirical evidence that patriotism can be elicited and leveraged to increase citizens’ trust and gain their voluntary cooperation with both the state and the community.

## Supporting information

Additional supporting information may be found in the online version of this article at the publisher's website:

Photos of fictional country and Austrian flags
Photos of Australian and Austrian national landscapes
