# Supplementary material for: Patriotism's Impact on Cooperation with the State: An Experimental Study on Tax Compliance
Source: Polit Psychol. 2015 Aug 28;37(6):867–81. doi: 10.1111/pops.12294 (PMC5125400; doi:10.1111/pops.12294)
Supplement: Supplementary file 1 — Photos of fictional country and Austrian flags Photos of Australian and Austrian national landscapes [file POPS-37-867-s001.pdf]

Appendix:

Materials for the flag manipulation

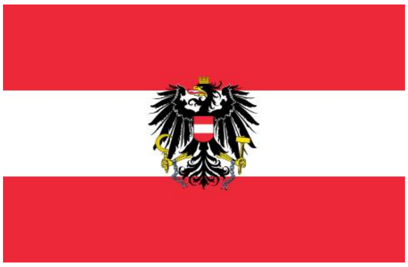

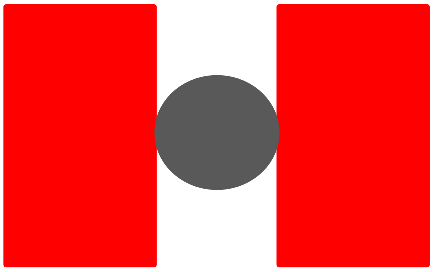

Materials for the national landscape manipulation

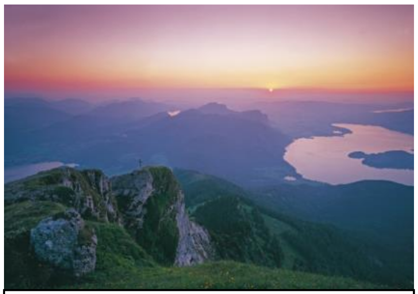

© Austria Tourism / Popp Hackner

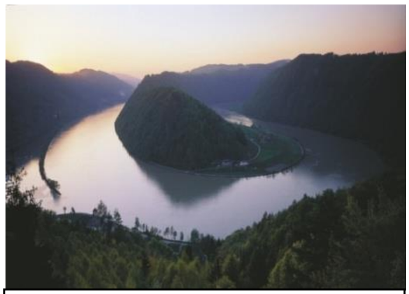

© Austria Tourism / Horvath

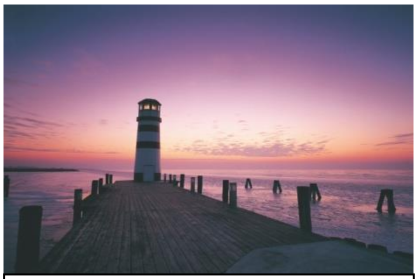

© Austria Tourism / Popp Hackner

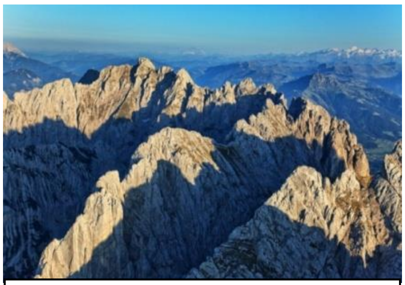

© Austria Tourism / Homberger

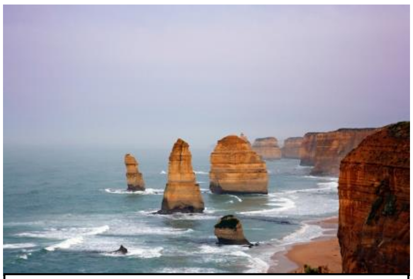

© Tourism Australia / Anonymous photographer

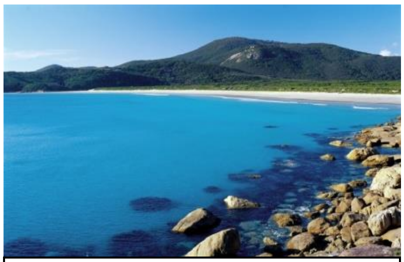

© Tourism Australia / Rains

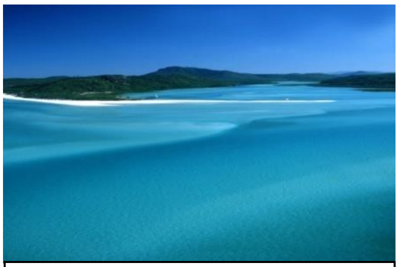

© Tourism Australia / Drewitz

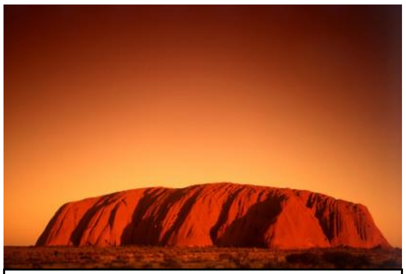

© Tourism Australia / Clarke
